# Outcomes of six dogs with prostate carcinoma and bacterial prostatitis treated with prostate artery embolization

**DOI:** 10.1093/jvimsj/aalag034

**Published:** 2026-03-06

**Authors:** Kornelia Tiffinger, Erin Gibson, Dana L Clarke, William T N Culp, Carrie Palm, Christopher Thomson

**Affiliations:** Department of Clinical Sciences and Advanced Medicine, University of Pennsylvania Matthew J. Ryan Veterinary Hospital, Philadelphia, PA 19104, United States; Department of Clinical Sciences and Advanced Medicine, University of Pennsylvania Matthew J. Ryan Veterinary Hospital, Philadelphia, PA 19104, United States; Department of Clinical Sciences and Advanced Medicine, University of Pennsylvania Matthew J. Ryan Veterinary Hospital, Philadelphia, PA 19104, United States; Department of Surgical and Radiological Sciences, School of Veterinary Medicine, University of California-Davis, Davis, CA 95616, United States; Department of Surgical and Radiological Sciences, School of Veterinary Medicine, University of California-Davis, Davis, CA 95616, United States; Department of Surgery, Veterinary Specialty Hospital—North County, San Marcos, CA 92064, United States

**Keywords:** interventional oncology, interventional radiology, intra-arterial antibiotics, radiology and diagnostic imaging, surgery, urogenital

## Abstract

**Background:**

Risk of recurrent lower urinary tract infection and bacterial prostatitis (BP) after prostate artery embolization in dogs diagnosed with concurrent BP and prostatic carcinoma (PC) is unknown.

**Hypothesis/Objectives:**

To report short- and long-term outcomes of dogs with PC and BP undergoing prostatic artery embolization (PAE).

**Animals:**

Six clients owned dogs with a concurrent diagnoses of BP and PC that subsequently underwent PAE and had a minimum follow-up of 4 months after PAE.

**Methods:**

Medical records of 6 dogs diagnosed with PC and BP and treated with PAE were retrospectively reviewed. Physical exams, clinicopathologic and imaging results, procedural details, and short- and long-term outcome data were evaluated.

**Results:**

Prostatic carcinoma was diagnosed via imaging findings (6/6), cadet-B-Raf protein (BRAF) testing (3/6), ultrasound-guided aspirates (2/6), and cystoscopic biopsy (1/6). BP was diagnosed based on clinical signs (6/6), imaging (6/6), urinalysis and positive urine culture (6/6), and concurrent positive prostatic wash cultures (2/6). All dogs received antibiotics based on urine culture and sensitivity testing (UCS) for a median of 7 weeks (range 2-13) before PAE. Two dogs had positive UCS at the time of PAE. One dog developed a prostatic abscess and 2/6 had positive UCS after PAE. Median survival was 13 months (4.5-17), and no dog died from infection.

**Conclusions and clinical importance:**

Urinary tract infection, BP, or a combination of both occurred in a minority of dogs undergoing PAE in this cohort and were not life-limiting complications. In dogs, concurrent BP and PC should not prevent treatment with PAE.

## Introduction

Prostate carcinoma (PC) is a locally aggressive and highly metastatic cancer occurring more commonly in older, neutered male dogs.^1,2^ Current local and systemic therapies appear to prolong survival but ultimately are unable to provide a cure.^3–7^ PC might predispose the prostate to bacterial colonization and infection through structural compromise by neoplastic transformation. Dogs with PC might develop bacterial prostatitis (BP), often secondary to ascending infection with aerobic bacteria such as *Escherichia coli*.^8–13^ The presence of BP in dogs with PC might complicate diagnosis of PC and can further delay anti-cancer care in these dogs which could lead to poorer outcomes compared to dogs without concurrent BP.

Prostate artery embolization (PAE) is a minimally invasive procedure to treat benign or malignant prostatic disease. It has been evaluated for feasibility, effectiveness, and short-term outcome in research dogs with induced benign prostate hyperplasia (BPH) ^14^ and for naturally occurring PC,^15–17^ although none report on the effect of concurrent lower urinary tract infection (UTI), if documented. Dogs who underwent PAE or prostate artery chemoembolization for naturally occurring PC demonstrated a decrease in prostate volume and improvement of clinical signs in short-term follow-up.^16–17^ These cumulative data to date support PAE as an effective and minimally invasive therapy for PC. Currently there are no clear guidelines for pre-PAE procedural care, but it is generally recommended to perform urinalysis, aerobic urine culture, and sensitivity testing to investigate for concurrent infection.^18^ Logically, the presence of infection in the prostate before PAE would increase the risk of complications such as abscessation/rupture or recurrent UTI due to the attenuation of blood supply to the prostate after the procedure, although this has not been investigated previously.^10,18^ Optimal delivery, duration, and type of antibiotic in dogs with concurrent UTI or BP and PC undergoing PAE are unknown.

The aim of this study was to describe the short- and long-term outcomes of dogs diagnosed with BP and PC who subsequently underwent PAE, and to describe the risk of prostatic abscessation and recurrent UTI after PAE within this cohort. The authors also aimed to summarize antimicrobial usage in this cohort in order to inform future treatment recommendations.

## Materials and methods

### Study design and inclusion criteria

Data were collected retrospectively from multiple institutions and analyzed descriptively to summarize clinical findings and outcome. The medical records were collected and reviewed from 2 universities and 1 private practice between November 2020 and June 2024. Dogs were included if they had a diagnosis of concurrent BP and PC, subsequently underwent PAE, and had a minimum follow-up of 4 months after PAE. The diagnosis of PC was based on cytology, histopathology, cadet-BRAF mutation test (Antech Diagnostics, Inc.) or a combination thereof. Diagnosis of BP, determined by the appearance of the prostate on imaging (ultrasound or CT angiography, CTA) and prostate wash cytology and culture, or urine culture was also required. Data recorded from the medical records included signalment (age, sex, breed, body weight), history, presenting complaints, results of diagnostics including bloodwork, urinalysis, urine culture, prostatic wash, fine-needle aspirates (FNA), abdominal ultrasound, radiographs of the thorax and abdomen, CTA, and cadet-BRAF mutation test. Medical treatments before and after PAE such as use of antibiotics, non-steroidal anti-inflammatory drugs (NSAIDs), steroids, chemotherapy, and radiotherapy were also recorded. Additionally, PAE procedure details and outcomes were recorded into a spreadsheet (Excel, version 1906, Microsoft Corp.). Descriptive statistics were generated, with median and ranges.

## Results

### Signalment and clinical presentation

A total of 6 dogs met the inclusion criteria. The median age was 11 years (range 8-11.8 years), and the median body weight was 26.3 kg (range 25-32 kg). There were 2 mixed breed dogs, and one each of the following breeds: Newfoundland, American pit bull terrier, Labrador retriever, and Miniature schnauzer. All dogs were neutered. Presenting complaints included mucopurulent hemorrhagic preputial discharge (2/6), stranguria (3/6), urinary incontinence (1/6), lethargy (1/6), tenesmus (2/6,) vomiting (2/6), diarrhea (1/6), and hyporexia (2/6).

### Pre-procedural diagnostics

The diagnostic tests performed were selected based on the attending clinician and owner preferences. CBC and serum biochemical panels were performed in all dogs. Hematological findings revealed neutrophilia in 2/6 dogs (median 10.94 × 10^3^ U/L; range: 3.17-11.07; ref. range analyzer A 2.7-9.4, analyzer B 3-10.5), mild anemia in 3 cases (median 38.2%, range: 35.7-52.3, reference range analyzer A 41-58, analyzer B 40-55), and for 1 dog CBC results were within reference intervals. Biochemical abnormalities included elevated Alkaline Phosphatase (ALP) (median 87 U/L, range:63-177, reference range analyzer A, 20-155, analyzer B 14-91) in 1/6 dogs, hypercholesteremia in 2/6 (median 264 mg/dL, range: 212-432, reference range analyzer A 128-317, analyzer B 139-353), elevated total protein (median:7.1 g/dL, range: 5.4-7.8, reference range analyzer A 5.4-7.1, analyzer B 5.4-6.9), total hypocalcemia (median:10 mg/dL, range: 9.4-10.3, reference range analyzer A 9.8-11.7, analyzer B 9.6-11.2), and hypoalbuminemia (median 3.1 g/dL, range: 2.8-4.1, reference range differed according to the analyzer A: 2.5-3.7 and 3.3-4.1 for analyzer B).

Biochemistry panels for 2/6 dogs showed no abnormalities. Urinalysis was performed in all dogs and identified the presence of leukocytes, RBCs, and microorganisms in all dogs at the time of diagnosis. All dogs underwent abdominal ultrasonography and CTA of the abdomen; 4 dogs also underwent a thoracic CT scan for staging.

Ultrasonography performed at initial evaluation showed abnormal prostatic features in all dogs including prostatic mineralization (4/6 dogs), cystic prostatomegaly with regional steatitis (4/6), periprostatic peritoneal effusion (1/6), and mild bilateral pyelectasia and medial iliac lymphadenopathy (2/6). CTA confirmed cystic prostatomegaly with mineralization in 5/6 dogs, sacral and internal iliac lymphadenopathy in 2/6 dogs, and no evidence of pulmonary metastatic neoplasia in 4/4 dogs.

Bacterial prostatitis was confirmed in 6 dogs before PAE based on the combination of ultrasound-guided fine-needle aspirates (FNA; 3/6 dogs) and cytology, microbiology testing including prostate wash (2/6) and culture (2/2), or urine analysis (6/6) and aerobic urine culture (6/6), paired with imaging findings.

Before PAE, organisms cultured included *B-hemolytic Streptococcus* (4/6 dogs) and *E. coli* (2/6), while one dog had a co-agent infection documented of methicillin-resistant *Staphylococcus pseudintermedius* and multidrug-resistant (MDR) *E. coli* (1/6). At the time of PAE, 4/6 dogs had negative urine cultures, and 2 dogs had persistent positive urine or prostatic wash cultures.

Four dogs had Cadet-BRAF tests submitted, of which 3 were positive. Two dogs underwent prostatic wash, with both demonstrating septic suppurative inflammation and concurrent epithelial atypia. One dog underwent cystoscopic biopsy of the prostatic urethra and had a histologic diagnosis of urothelial/PC.

### Clinical management and outcome

Preoperatively, all dogs were administered NSAIDs, including carprofen in 2/6 dogs (median dose 1.8 mg/kg, range 1.5-2.1 PO q12h), piroxicam in 2/6 (median dose 0.28 mg/kg, range 0.28-0.29 mg/kg PO q24h), and meloxicam in 2/6 dogs (0.1 mg/kg PO q 24h). In addition, all dogs received appropriate antibiotic therapy based on urine culture and sensitivity testing (UCS) before PAE. The median antibiotic treatment duration before PAE was 7 weeks (range 2-13). Antibiotics included enrofloxacin in 4/6 dogs (median dose 6.2 mg/kg, range 5-9.8 mg/kg PO q24h), trimethoprim/sulfadiazine in 2/6 (median dose 19.5 mg/kg, range 18-21 mg/kg PO q12h), clindamycin in 1 dog (12 mg/kg PO q12h), meropenem in 1 dog (1 mg/kg SC q12h), and amoxicillin trihydrate/clavulanate in 1 dog (17 mg/kg PO q12h). All dogs underwent general anesthesia, with the anesthetic protocols individualized for each dog by a board-certified anesthesiologist. Five dogs received antimicrobials intravenously during anesthesia, including cefazolin in 3/6 (22 mg/kg intravenous [IV] q 90 minutes) and ampicillin sodium/sulbactam sodium in 2 (22-30 mg/kg IV q 90 minutes). One dog received meropenem (6 mg/kg intra-arterially in each prostate artery for a total dose of 12 mg/kg) at the time of PAE. Each PAE was performed by clinicians with extensive interventional radiology experience through the carotid artery as previously described.^16^ Embolization was considered complete after embolic microspheres (Bead Block; Boston Scientific Corp; 100-300 μm in size) were delivered intra-arterially into the tumoral blood supply to apparent stasis. In 1 dog, the right prostatic artery could not be accessed due to tortuous vascular anatomy, and 2 embolization coils (Hilal, 0.5 cm long, straight) were introduced into the left prostatic artery to allow for complete embolization with microspheres, while preventing non-target embolization to the caudal vesical artery.

In the dog receiving intra-arterial antibiotics, after super selection with the microcatheter and before delivery of embolic microspheres, half of the calculated antibiotic dose was delivered into one prostatic artery, and this procedure was repeated in the contralateral artery.

No major perioperative complications were recorded, and no evidence of non-target embolization was noted in any of the patients. After PAE, all dogs were observed to urinate and empty their bladder before discharge.

After PAE, 3 dogs developed lower UTI (*n* = 2) or prostatic abscessation (*n* = 1). Two of these dogs had a single infection 5 and 6 months after PAE, with the same bacteria cultured before PAE. Antimicrobial treatments were prescribed based on UCS and included amoxicillin/clavulanate acid (13.5 mg/kg PO q12h) in one dog and enrofloxacin (9.7 mg/kg PO q24h) in the other. The third dog was diagnosed with prostatic abscessation based on positive urine culture, pyuria, and progressive right prostate lobe cavitation with reactivity including regional peritonitis and static mild right medial iliac lymphadenopathy 6 weeks post-PAE. Repeat cultures consistently identified non-hemolytic *E. coli*. Medical management with culture-directed antimicrobials was elected over percutaneous abscess drainage or surgery due to the associated risks and good clinical status of the dog for the subsequent 4 months. Antibiotic administration was discontinued despite positive urine culture based on ultrasound findings of reduced echogenicity of the cavitary fluid, reduced peri-prostatic steatitis, and sustained resolution of signs.

Other treatments after PAE included NSAIDs alone (2/6) or in combination with IV or intraarterial (IA) chemotherapy (4/6) including piroxicam (0.28-0.29 mg/kg PO q24h) in 2/6, carprofen (2-2.2 mg/kg PO q12) in 2/6, and meloxicam (0.1 mg/kg PO q 24h) in 2/6. Mitoxantrone (3.65-5.5 mg/m^2^, q2-3 weeks IV) was delivered post-PAE in 3 dogs. Carboplatin (280 mg/m^2^ q4-weeks IV) and carboplatin (250 mg/m^2^, twice in 5-week intervals IA) were delivered in one dog each. One dog was transitioned to prednisone (1 mg/kg PO q24) due to poor tolerance of NSAIDs. Another dog also underwent stereotactic radiation therapy (GY 10 × 3 fractions) to the prostate.

The median survival time (MST) for all dogs was 13 months (range 4.5-17). Euthanasia was performed in 5/6 cases due to evidence of disease progression including local progression and ureteral obstruction in one dog and distant metastatic disease in 4 dogs, including vertebral and pelvic bone (2/6 dogs), lung (1/6), and ocular (1/6) metastasis.

## Discussion

The dogs in this report with concurrent BP and PC treated with PAE had largely positive outcomes. With appropriate prescription of antimicrobials based on UCS, recurrent UTI, BP, or a combination was infrequent. All dogs with UTIs, BP, or a combination after PAE were successfully medically managed, and procedural outcomes were good. The overall MST of 13 months was relatively long, and no dog had any life-threatening complications secondary to UTI or BP. In addition, death secondary to local disease progression was uncommon (1/6 dogs).

In dogs, the most frequently isolated organism in prostatic infections is *E. coli* with other common bacteria including *Staphylococcus* spp., *Streptococcus* spp., *Proteus mirabilis, Klebsiella* spp., *Mycoplasma canis*, and *Pseudomonas* spp.^19–21^ The dogs in this study were infected by organisms reported to be common in BP including *B-hemolytic Streptococcus* and *E. coli*. Several dogs developed MDR infections that appeared recurrent or persistent in the face of appropriate administration of antibiotics. Challenges in eradicating infection might result from the altered architecture and cavitations frequently accompanying neoplastic changes to the prostate, changes in prostatic fluid composition and loss of normal physiological mechanisms that prevent bacterial colonization in health,^21^ and the diminished therapeutic effect of the appropriate antimicrobial treatment in cases with MDR bacteria.^22–23^

At the time of PAE, 4/6 dogs had negative urine cultures, while 2/6 receiving appropriate antibiotic therapy based on UCS at the time of PAE still had persistent positive urine or prostatic wash cultures. In this cohort, the 2 dogs with positive urine cultures at the time of PAE did not have recurrent BP, lower UTI, or a combination documented after the intervention. It is unknown why the infection cleared in these 2 dogs after PAE; however, it is possible that the use of perioperative antimicrobials, as well as the concurrent use of IA antibiotics in one dog might have contributed to ongoing infection control. Alternatively, the reduction in tumor volume typical of this procedure could have helped reduce susceptibility to recurrent infections.

Of the 4 dogs with negative cultures prior to PAE, only 2 developed lower UTI or prostatic abscessation, and both were successfully medically managed. One dog for which a recurrent MDR *E. coli* infection after PAE was documented, successful embolization was only possible unilaterally; that dog received corticosteroids for 3 months due to intolerance of NSAIDs and received coils in addition to beads for embolization. In humans, both splenic embolization with coils^24^ and failure to provide intra-arterial antibiotic prophylaxis at the time of splenic embolization, regardless of embolic, appears to increase susceptibility to infection.^25^ It is possible that the use of coils, corticosteroids, or both in this dog increased susceptibility to infection. Additionally, embolization of one lobe of the prostate rather than both could have prevented substantial tumor response to diminish susceptibility to recurrent infection. Due to the retrospective nature of this report, tumor volume was not routinely measured and reported in all patients before and after PAE and therefore it is difficult to draw conclusions in this regard beyond speculation. However, the prolonged MST and technical procedural success within this cohort suggest that PAE is a reasonable treatment for PC in dogs with PC and BP.

Optimal antibiotic duration in human and veterinary medicine before PAE remains unclear. Alrashidi et al. reported intraprostatic abscess formation in people post-PAE for BPH, even with 2 sterile urine cultures before the procedure. It was hypothesized that ischemic effects and residual organisms in the prostate gland were contributory factors to infections diagnosed after treatment, and it is recommended to delay PAE for at least 2 months after consecutive sterile cultures and to administer long-term antibiotics.^26^ Based on these recommendations, none of the dogs of this report would have been considered optimal embolization candidates; however, these dogs still had good short- and long-term results. Additionally, delaying treatment of PC for 2 months beyond a negative culture is not ideal in a cancer control setting. Differences in disease process and species might account for differences in susceptibility to infection post-PAE.

In human medicine, PAE is primarily performed for benign prostatic hyperplasia. Conversely, in veterinary medicine, PAE is performed exclusively for PC. At the time of diagnosis, canine PC demonstrates high rates of local invasion and significant regional (15-72%) and distant (8-50%) metastasis with over 80% of dogs exhibiting gross metastatic disease at the time of death.^3,27–29^ Reported MSTs for dogs with PC are 3-18 months, even with combination therapy such as NSAID, radiation, chemotherapy, and surgery, or just surgery.^5,7,29–31^ In addition, in many veterinary patients, the development of life-threatening urethral or ureteral obstruction might lead to decisions for euthanasia. Given this, the risk of delaying treatment and following the human guidelines above must be weighed carefully in our veterinary population. It remains unclear how important a negative culture is at the time of PAE, as long as appropriate UCS-guided antimicrobial treatment is instituted throughout the peri-procedural time. Given the infrequent recurrence of infection observed in this cohort of dogs, it is also possible that treatment aimed at reducing tumor burden also could diminish susceptibility to BP or UTI.

In this case series, one dog received antibiotics IA before PAE during the same anesthetic event. The effects of antibiotic agents IA for the prevention of prostatic abscess and infection formation have not been investigated previously. While the pharmacokinetics of antibiotics delivered IA into the prostatic arteries before embolization has not been investigated, previous studies have identified a potential protective role of delivering antibiotics IA before tissue embolization in diminishing risk of post-procedural abscessation. Antibiotic treatment IA is useful in preventing inflammatory reactions and infection after partial splenic embolization in people. Antibiotics administered IA during splenic embolization are associated with fewer splenic abscesses.^24^ Additionally, successful treatment of hepatic abscesses with antibiotics IA, when percutaneous drainage could not be performed and IV administration of antibiotics was ineffective, is reported.^32–33^ The published experience in human medicine of IA antibiotics in embolization procedures, while infrequent, appears to support a diminished rate of post-procedure infection and could be considered for PAE procedures with or without documentation of BP before treatment.

Two dogs in this report received chemotherapy IA for PC treatment. One dog received chemotherapy IA before PAE, and another received chemotherapy IA after PAE to treat regional lower urinary tract metastasis. In dogs with naturally occurring lower urinary tract tumors, chemotherapy IA has been compared to chemotherapy IV regarding tumor response. The chemotherapy IA group had significantly smaller tumor size in the longest unidirectional measurement and these dogs were significantly more likely to achieve tumor response compared to the chemotherapy IV group.^34^ While the role of chemotherapy IA for canine PC is unknown, the authors consider it a reasonable stand-alone palliative treatment or as a treatment in dogs with BP and PC, while concurrent antimicrobial treatment is undertaken before PAE.

The limitations of this study include its retrospective design, as well as the multi-center approach to accruing cases. This approach might introduce variability in how data were reported and in the overall treatments or recommendations offered to owners. Additionally, only limited cases were included due to the infrequent presentation of this clinical syndrome and the early usage of the PAE procedure.

## Conclusion

These data suggest that PAE is a valuable treatment option for dogs diagnosed with BP and PC concurrently and that dogs can have positive outcomes after PAE treatment. Careful preoperative antibiotic treatment based on UCS testing and close postoperative monitoring for recurrence of infection is crucial to optimize long-term outcomes. While the risk of post-PAE infections such as prostatic infection, abscessation, and UTI exists, total resolution of BP is possible and occurred in most cases within this cohort. IA antibiotics were rarely provided in this cohort, so it is difficult to make conclusions about their utility in this patient study. Given the high safety profile of IA antibiotics and the potential benefits, they could be considered in future similar cases. Attempting to resolve BP or UTI before PAE is ideal and recommended; however, based on the positive outcome of the dogs presented in the study, significantly prolonging time until treatment with PAE should be avoided.

## Off-label antimicrobial declaration

The authors declare no off-label use of antimicrobials.

## Institutional animal care and use committee or other approval declaration

The authors declare no institutional animal care and use committee or other approval was needed.

## Abbreviations

PC: prostate carcinoma
BP: bacterial prostatitis
PAE: prostatic artery embolization
UCS: urine culture and sensitivity testing
UTI: urinary tract infection
BPH: benign prostate hyperplasia
CTA: computed tomography angiography
FNA: fine needle aspirates
NSAID: non-steroidal anti-inflammatory drugs
CBC: complete blood count
MRSP: methicillin-resistant *Staphylococcus pseudintermedius*
MDR *E. coli*: multidrug-resistant *E. coli*
PO: per os
IV: intravenous
IA: intraarterial
